# Rheological and Nutritional Characterization of Sweet Corn By-Product (Cob) to Develop a Functional Ingredient Applied in Dressings

**DOI:** 10.3389/fnut.2021.666654

**Published:** 2021-04-28

**Authors:** Sandra Castillo, Alexis Rodríguez, Minerva Bautista-Villarreal, Nallely García-Solano, Claudia Gallardo-Rivera, Juan G. Báez-González, Eduardo Sánchez-García, Karla G. García-Alanis

**Affiliations:** ^1^Departamento de Alimentos, Facultad de Ciencias Biológicas, Universidad Autónoma de Nuevo León, San Nicolás de los Garza, Mexico; ^2^Departamento de Química, Facultad de Ciencias Biológicas, Universidad Autónoma de Nuevo León, San Nicolás de los Garza, Mexico

**Keywords:** by-product, bromatological, cob, food waste, functional ingredient, sustainable industry

## Abstract

In this study, a flour from corn cob (central core of the maize ear, stage R4) was obtained through three treatments. The three flours obtained were characterized by bromatological analysis, yield, and granulometry. Additional dressing-type oil in water (O/W) emulsions were developed, varying the formulation by incorporating distinct amounts of corn cob flour. The formulations' stability was evaluated over a period of 21 days, determining the particle size, creaming index, coalescence rate, consistency coefficient (*k*), and flow behavior indices (*n*). Results have shown significant differences in protein, fat, and carbohydrate content in the flour, depending on the cooking treatment. A good percentage of grinding yield was obtained (98%), in addition to several fractions by granulometry (60, 120, 250 MESH), showing differences in their nutritional content. Finally, the particle size of O/W emulsions developed varied among formulations. The combination of 0.6% of xanthan gum (XG) and corn cob flour showed major stability in average droplet size. No significant differences were observed in the coalescence rate values for the three formulations. Still, significant differences in the creaming index were evidenced in those formulations without XG or corn cob flour. The results regarding the consistency coefficient (*k*) and flow behavior indices (*n*) suggest a possible synergy between XG and flour of corn cob for enhancing the viscosity and pseudoplasticity of dressings in a concentration-dependent manner.

## Introduction

The growing demand for food worldwide has led to a large amount of organic waste, including vegetable residues. Their production and handling generate greenhouse gas (GHG) emissions during their collection, transport, and deposit in landfills (1, 2). Given the global need to advance toward sustainable productivity and consumption due to the food waste and losses, which concern around the world, the development of alternatives for the possible uses of food waste in the industry is an urgent need (3). Maize, being one of the most widely-consumed foods, is grown in large quantities around the world. Corn, prepared in different ways is widely consumed by humans in Latin America, Africa, and the Balkans (4). Traditional corn food varies worldwide, and Mexico is one of the major producers and consumers of this product. Corn products such as sopes, tortillas, and champurrado are examples of Mexican food made from maize and are available in many markets. With respect to this type of crop, only in Mexico, 60,270 tons of sweet corn are produced per year (Stage R3–R4), generating ~10,000 tons of residue. The cob is given as a residue or agricultural by-product in large quantities, the grain being separated from the cob (R6). It is estimated that for each ton of corn, 170 kg of cob is obtained (5). Although this waste is used as feed in livestock systems, a large amount goes to landfills (6). The use of chemical application of cob has been significantly restricted due to the difficulty that exists to access its components (recalcitrant character), the incomplete chemical characterization, and the evaluation of its main products (lignin, cellulose, and hemicelluloses) (7). Due to this situation, various studies have been directed toward obtaining enzymes, bioconversions through fermentation, or cellulose and/or xylo-oligosaccharides. Still, there are few studies of its chemical characterization; also, there are no studies specifically focused on its characterization as a possible functional ingredient and its rheological properties.

On the other hand, dressings are oil-in-water (O/W) emulsions in which the mixture of different ingredients (vegetable oil, egg yolk, xanthan gum, sugar, salt) promote stability and help enhance its organoleptic characteristics (8, 9). There is currently a wide variety of products such as mayonnaise or dressings, which should contain more natural ingredients, avoiding the addition of chemicals or animal origin-ingredients. Likewise, the development of products with more desirable nutritional value (fiber, protein, minerals, vitamins, etc.) is chosen due to the effort toward clean labeling. This implies the performance of efficient product monitoring mechanisms that ensure their quality, in addition to the knowledge of their physicochemical characteristics (9). With respect to everything mentioned above, the objective of this work was to obtain and characterize a cob flour for its possible application as a functional ingredient in dressings that could provide added value to the product and contribute to sustainable use of food.

## Materials and Methods

### Biological Material

The whole corn cobs in stage R4 (immature) were purchased from retail markets in the metropolitan area of Monterrey, Nuevo León, Mexico. They were identified in the Department of Botany, Universidad Autónoma de Nuevo León. Whole plant material was washed in order to remove any visible dirt on the shell. After that, they underwent three types of treatment (process) to obtain three types of flour.

#### Flour From Treatment 1 (FT1)

The shell and kernel were separated by hand with a knife from the cob (central core of maize). The cobs were cut crosswise into 2 mm of thick pieces and were dried (raw material) at 45°C/24 h in a convection oven (Model HS, Riossa). After that, they were ground in a mill with a mesh of 1 mm (Thomas Wiley, Model 4) according to manufacture instructions. Since this flour (FT1) was obtained from the plant material without any cooking treatment, it was taken as a control as a point of comparison of the resulting nutrients with those that had been cooked.

#### Flour From Treatment 2 (FT2)

The shell and kernel were separated by hand with a knife, from the cob. The cobs were cooked in a cooking pot (10 L) with water at boiling temperature (96 ± 2°C) for 3 h. After that time, the water was drained, the cobs were then cut crosswise into 2 mm thick pieces and dried at 45°C/24 h in a convection oven (Model HS, Riossa). They were then ground in a mill with a 1 mm-mesh (Thomas Wiley Model 4) according to manufacturer instructions.

#### Flour From Treatment 3 (FT3)

Only the shell was separated from the cob (by hand), the whole corn cob was cooked in a cooking pot (10 L) with water at boiling temperature (96 ± 2°C) for 3 h. After that time, water was drained, the grain was removed, cobs were cut crosswise into 2 mm thick pieces and dried at 45°C/24 h in a convection oven. After that, they were ground in a mill with a 1 mm-mesh (Thomas Wiley Model 4).

The flours obtained from the three treatments (FT1, FT2, FT3) were stored in clean and dry jars for further analysis. The cooking water was kept in closed sterile jars in order not to generate residues as much as possible. It was frozen at −20°C until use.

### Bromatological Analysis

Bromatological analyses were conducted on the flours obtained in each treatment. Moisture content, ash, protein, fat, total carbohydrates and raw and dietary fiber were determined using the methods described by the Association of Official Analytical Chemists (10). Moisture: Dehydration in an oven at 105°C; ash: combustion at 450°C for 12 h; crude protein: using the method of Macro Kjeldahl, (Nx6.25); fat: ethereal extraction method (AOAC 972.28); total carbohydrates: (AOAC 923.03); crude fiber: (AOAC 985.29). Dietary fiber: Enzymatic-gravimetric method (AOAC 991.43).

### Physical Analyses

The physical analyses, including grinding yield, mechanical granulometry, and rheology were carried out only in the FT3 because it shows the best results in bromatological analyses, yielding significantly most crude fiber and protein, the least fat and total carbohydrates, and the greatest amount of minerals.

#### General and Grinding Yield

The general yield, in addition to the grinding yield, was determined for the flour from the cob. The general yield was obtained by weight difference between cooked and dried shelled cob. The grinding yield was obtained to determine if there are losses during grinding. Briefly, 500 g of cobs obtained from treatment 3 (FT3) were ground in a mill, and the weight was registered before and after grinding. The grinding yield was calculated with the formula:

$$\begin{array}{l} grinding\;yield\;=100\;-\left[\frac{cob\;weight-flour\;weight\;}{cob\;weight}X\;100\right] \end{array}$$

#### Mechanical Granulometry

The term granulometry is used in the food industry to characterize some raw material's granularity, passing them through sieves of different sizes. These sieves should be organized in decrescent form while measuring their mass retained by each sieve (11). The FT3 (500 g) was passed through different sieves (#5, 40, 60, 120, and 250 MESH) for 10 min in constant vibration. The flour retained was weighed, and the percentage of retension in each sieve was calculated with the formula:

$$\begin{array}{l} \%\;retension\;of\;FT3\;=\;100-\left[\frac{total\;FT3-FT3\;retained}{total\;FT3}X\;100\right] \end{array}$$

A bromatological analysis was performed on the three different fractions obtained from sieves (60, 120, 250 MESH), facilitating the choice of the best option for the development of the functional ingredient. Based on the bromatological analysis results, # 250 mesh flour (FT3-250, see 3.0 results section) was selected for further analyses due to its higher protein content and its smaller particle size. Besides this fraction, the dietary fiber content was determined.

### Emulsion Preparation and Rheological Characterization

#### Emulsion Preparation

Emulsions (E) were developed to evaluate FT3-250 as a functional ingredient that does not cause destabilization of the system, and at the same time, provides added value. Three formulations (E1, E2, E3) were made, varying the percentage of: cooking water (38.3–39.1%), sugar (1.25–5.75%), xanthan gum (XG, 0–0.72%) Sigma–Aldrich (Sigma Chemical Co., St. Louis, MO, USA) and/or FT3-250 flour (0–4.5%) (**Table 5**). Briefly, the emulsifiers (egg yolk and xanthan gum) were hydrated with the cooking water and constantly stirred (500 rpm, IKA T50 Digital Ultra Turrax) for half an hour. After gel formation, the other ingredients (sugar, salt, and FT3-250) were added. Finally, the oil was added slowly in a continuous manner, allowing it to be incorporated into the mix. The mix was stirred for 3 more min. at 3,000 rpm and then 2 more min. at 500 rpm (12). Four variants (A, B, C, D) of each emulsion (E1, E2, E3) were obtained based on the amount of FT3-250 added (**Table 5**). The emulsions without the addition of FT3-250 were used as respective controls (E1-A, E2-A, E3-A). Each formulation containing xanthan gum (XG) and/or FT3-250 in different proportions was evaluated for droplet size, coalescence rate, cremation index, and rheological parameters [consistency coefficient (*k*) and flow behavior indices (*n*)].

#### Physical Emulsion Stability

##### Droplet Size and Coalescence Rate

The emulsions' droplet size measurements containing 0, 2.5, 3.5, and 4.5% of FT3-250, were monitored using a Malvern Mastersizer 3000 (Malvern Instruments, Ltd, Worcestershire, UK) particle size analyzer with a unit of Hydro LV with water as a dispersant. The angular scattering intensity data were analyzed to calculate the particles' size, creating a scattering pattern using the Mie theory of light scattering. The software calculated the particle size distribution as Sauter mean diameter (d_3,2_). The optical properties of the sample were defined as a refractive index of 1.460 with absorption of 0.1.

The coalescence rate exhibits first-order kinetics (13, 14) and is expressed with the formula:

$$\begin{array}{l} \frac{Nt}{No}=\;e^{-k}c^{t} \end{array}$$

Where: *Nt* is the concentration in the number of drops at a time (*t*), *N*_0_ is the concentration in the number of the newly formed drops (time = 0), and K_c_ is the rate constant, which is related to the probability that the interfacial film will break at a certain time (*t*). The volume of the emulsion droplets remains constant when there is nooil release in the emulsion.

##### Creaming Index

To evaluate the emulsion stability against creaming, the method mentioned by Ye et al. (13) and Klinkesorn et al. (15) with minor modifications were carried out. The emulsions were placed in glass tubes, filling up to 10 cm and stored at 3.5°C for 24 days. The measurement of the height of the opaque layer (H_S_) and total height (H_E_) were determined at the end of storage and calculated with the following equation:

$$\%\;Creaming\;index=[\;\frac{Hs}{HE\;}\;]\;X\;100$$

Where *H*_*S* =_ Height of opaque layer and *H*_*E* =_ Total height (15).

#### Rheological Parameters (*k* and *n*)

A rotational test was performed using a ReolabQC rheometer (Anton Paar, Graz, Australia) to measure rheological parameters. The consistency coefficient (*k*) and flow behavior indices (*n*) of the developed emulsions were obtained as a function of the shear rate (1–100 s^−1^) with CC-27 geometry at a temperature of 25°C (16).

### Statistical Analyses

All measurements were performed in triplicate, and ANOVA was performed with a confidence level of 95% (*p* < 0.05) using SPSS 20 software (IBM, SPSS Inc., Chicago, IL, USA). To determine the statistically significant difference between values, one-way variance analysis and a Tukey test were performed.

## Results

### Bromatological Analyses of the FT1, FT2, and FT3

Three different flours from corn cobs were obtained. Two of them were derived from different cooking treatments (FT2 and FT3). The other one, without cooking treatment (solid-state, stage R4), was used as control (FT1). These were analyzed for bromatological composition. Results are presented in Table 1. The results showed that the corn cob in solid-state (stage R4, without cooking: FT1) is a by-product rich in fiber (12%) and total carbohydrates (81.63%), which additionally contains around 2.42% protein and (ash-2.58%) total minerals, in addition to containing 1% fat. The cooking treatment (FT2 and FT3) applied to the cob samples significantly (*p* ≤ 0.05) decreases the content of fat (0.90–0.53%), total carbohydrates (77.14–74.03%), and at least in FT2 (cob cooked without grain), the total mineral content (1.66 %). On the other hand, cooking treatment significantly (*p* ≤ 0.05) increases the protein (4.67–5.03%) and crude fiber content (14.34–17.61%) in both flours (FT2 and FT3, respectively, Table 1).

**Table 1:** Bromatological content (%) of three different flours from corn cob (FT1, FT2, FT3).

	**Sample**					
**Parameter**	FT1 Control^*^		FT2^*^		FT3^*^	
	**Moist matter**	**Dry Matter**	**Moist matter**	**Dry Matter**	**Moist matter**	**Dry Matter**
Moisture	1.32 ± 0.39	0	1.16 ± 0.07	0	2.76 ± 0.18	0
Ash	2.54 ± 0.04	2.58 ± 0.04^a^	2.28 ± 0.8	1.66 ± 0.07^b^	1.61 ± 0.07	2.30 ± 0.08^a^
Fat	1.20 ± 0.10	1.22 ± 0.10^a^	0.90 ± 0.04	0.93 ± 0.03^b^	0.53 ± 0.10	0.54 ± 0.11^c^
Protein	2.39 ± 0.06	2.42 ± 0.07^b^	4.89 ± 0.43	5.03 ± 0.44^a^	4.62 ± 0.30	4.67 ± 0.30^a^
Crude Fiber	12.00 ±1.16	12.16 ±1.13^c^	14.92 ± 0.95	15.34 ± 1.00^b^	17.42 ± 0.90	17.61 ± 0.90^a^
Total Carbohydrates	80.54 ± 0.92	81.63 ± 1.21^a^	74.94 ± 1.24	77.14 ± 1.32^b^	74.03 ± 0.99	74.87 ± 1.00^b^

* *Values given are averages of three replicates ± standard deviations. Data were analyzed by an analysis of variance test (one-way ANOVA) and a post hoc test of Tukey's multiple range. Means within a row which are not followed by a common superscript letter (a, b, c) are significantly different (p ≤ 0.05)*.

### Physical Analyses

#### General and Grinding Yield

Two different yields were determined only for the FT3 due to the bromatological characteristics (low fat content, high protein content, minerals and crude fiber). Table 2 shows the average of general and grinding yield. The general flour yield (FT3) obtained from cob was 40.27 ± 0.57% on average since, during the drying process, it loses most of its weight in the form of water. On the other hand, the grinding yield was 98.26 ± 0.65, indicating that there is little loss during the grinding process. Sample losses in standard grinding processes are due to different factors, such as poor sample conditioning, the type of mill sieves, or grinding energy, among others (17).

**Table 2:** General and grinding yield of the FT3.

**Flour**	General yield^*^ (%)	Grinding yield^*^ (%)
FT3	40.27 ± 0.57	98.26 ± 0.65

* *Values given are averages of three replicates ± standard deviations*.

#### Granulometry

The granulometry of samples helps to determine the use that will be given to them, depending on the particle size that is required for a specific area, which is also is associated with the necessary quality parameters in flours for the development of new products (11, 18). The FT3 was passed through different sieves (#5, 40, 60, 120, 250 MESH).Weights of the material retained in each were registered as a percentage of retention. The retained material in each sieve was taken as a fraction and was associated with the particle size corresponding to the sieve mesh size. Table 3 shows the percentage of FT3 retained in each sieve. Four fractions were obtained (FT3-40, FT3-60, FT3-120, FT3-250) in size ranges of 53–400 μm. A higher percentage of flour retention is observed in the # 40 MESH sieve (400 μm) with 62.68%, followed by #60 mesh (22.81%), # 120 mesh (7.67% ), while the lowest retention percentage is observed in the # 250 MESH sieve (53 μm) with 6.81%. To verify differences in each fraction's composition, a bromatological analysis was carried out (see section Bromatological Analysis) in three of the four fractions obtained (FT3-60, FT3-120, FT3-250). These fractions were selected because they had a smaller particle size. Results are presented in Table 4. According to the results, the content of ash did not vary among the fractions. The moisture percentage in the flours FT3-250 and FT3-60 are statistically equal (4.02 and 4.06%, respectively), whereas the FT3-120 showed a higher quantity (4.54%). The smallest particle size fractions (FT3-250, FT3-120) presented a significantly higher amount of protein (4.87 and 4.31%, respectively), crude fiber (36 and 28%, respectively), and a lower amount of total carbohydrates (57 and 66%, respectively) than FT3-60 (Table 4).

**Table 3:** Percentage of FT3 fractions retained on each sieve.

**Sieve (#Mesh)**	**Size (μm)**	**Retention (%)**	**Fraction**
5	4,000	0^*^	–
40	400	62.70 ± 0.83	FT3-40
60	250	22.81 ± 1.13	FT3-60
120	125	7.67 ± 0.44	FT3-120
250	53	6.81 ± 0.5	FT3-250

* *Values given are averages of three replicates ± standard deviations*.

**Table 4:** Bromatological analyses of the FT3 fractions obtained in different sieves.

**Parameter**		**FT3-250**		**FT3-120**		**FT3-60**	
		**Moist matter %**	**Dry matter %**	**Moist matter %**	**Dry matter %**	**Moist matter %**	**Dry matter %**
Moisture		4.02 ± 0.06^a^	0^*^	4.54 ± 0.15^b^	0^*^	4.06 ± 0.14^a^	0^*^
Ash		1.53 ± 0.28	1.59 ± 0.29^a^	1.50 ± 0.43	1.57 ± 0.45^a^	1.11 ± 0.30	1.16 ± 0.31^a^
Protein		4.67 ± 0.18	4.87 ± 0.19^a^	4.11 ± 0.33	4.31 ± 0.35^a^	2.87 ± 0.09	2.99 ± 0.09^b^
Crude fiber		34.70 ± 1.80	36.03 ± 1.87^a^	26.75 ± 0.48	28.00 ± 0.49^b^	19.98 ± 0.36	20.75 ± 0.39^c^
Total carbohydrates		55.08 ± 6.41	57.41 ± 6.65^a^	63.10 ± 0.47	66.12 ± 0.19^b^	71.98 ± 3.40	75.10 ± 3.65^b^
Dietary fiber	Soluble	2.44	2.54	ND	ND	ND	ND
	Insoluble	73.87	76.96	ND	ND	ND	ND
Avilable carbohydrates		13.47	14.04	–	–	–	–
Total sugars		6.76 ± 0.44	7.04 ± 0.46_a_	8.27 ± 0.16	8.66 ± 0.16^b^	6.21 ± 0.06	6.47 ± 0.06^a^

* *Values given are averages of three replicates ± standard deviations. Means within a column which are not followed by a common superscript letter (a, b, c) are significantly different (p < 0.05). FT3-250 (#250 mesh), FT3-120 (#120 mesh), FT3-60 (#60 mesh) ND, not determined*.

### Emulsion Preparation and Rheological Characterization

#### Droplet Size and Coalescence Rate

**Figure 1:**
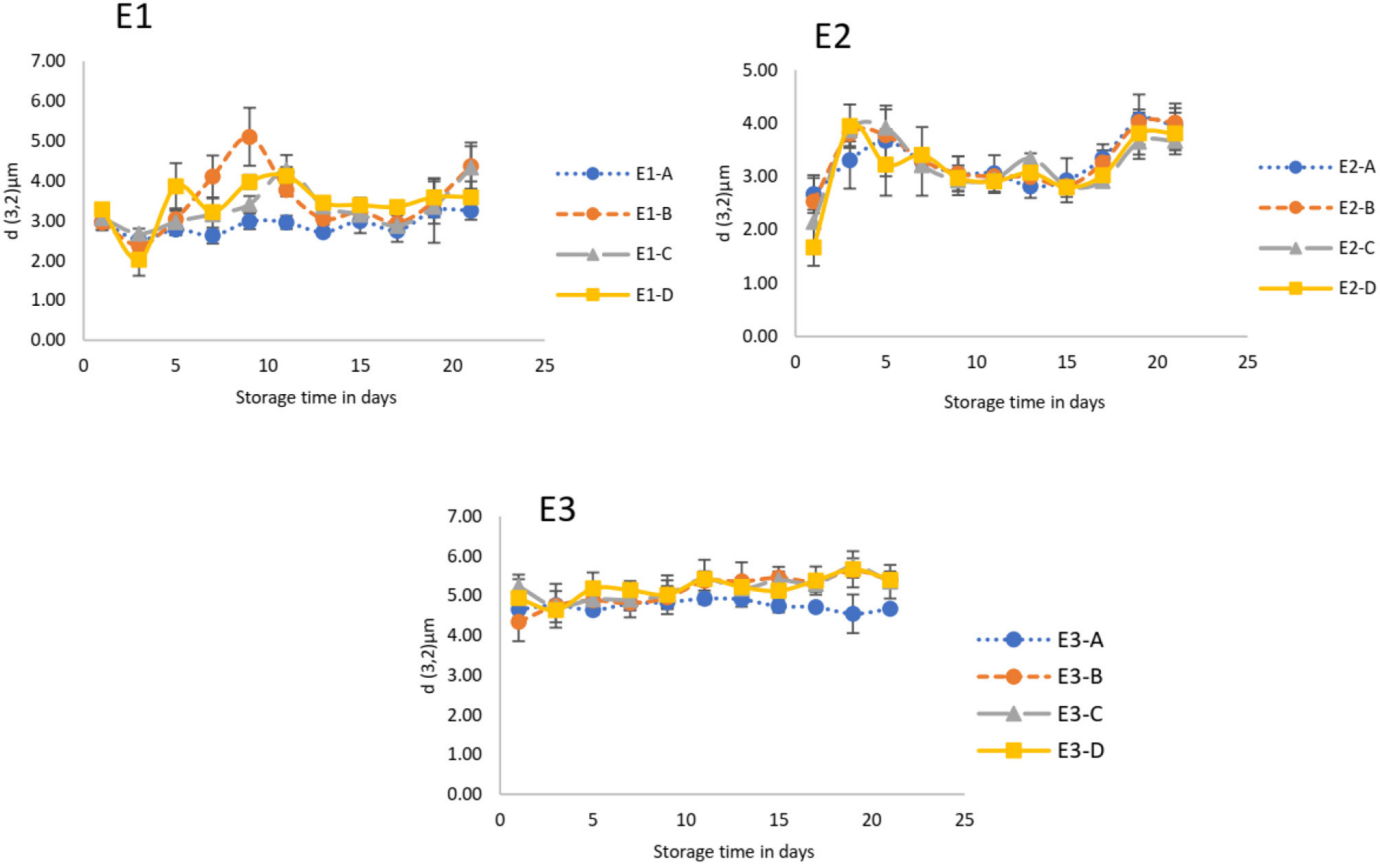
Influence of concentration of XG and/or FT3-250 on the emulsion's average droplet size (d_3,2_) as a function of storage time at 20°C. E1, E2, E3 (0.72, 0.36, 0% XG, respectively) and their variants (A = 0%, B = 2.5%, C = 3.5%, D = 4.5% of FT3-250). Mean values ± standard deviation (represented by bars).

Three different emulsions (E1, E2, E3) varying with respect to the proportion of XG and FT3-250 were developed (Table 5). These three emulsions had four variants (A, B, C, D, E). These last variations are a function of the amount of FT3-250 and are described in Table 5. The droplet sizes (d_3,2_) of each emulsion were measured in time (21 days) at 20°C. Time-dependent changes in the average droplet diameters (d_3,2_) of emulsions (E1, E2, E3) containing different concentrations of XG and/or FT3-250 are shown in Figure 1. In the graph, more homogeneous behavior in terms of droplet diameteris observed in E2 and E3. No significant differences were found in diameters (d_3,2_) in formulations E2 and E3 during the storage period compared to their respective control. On the other hand, the E1 shows a variable behavior for each inclusion degree FT3-250. Despite the variability in the behavior compared to the control (E1-A), there was a significant difference (*p* ≤ *0.05*) only on the tenth day of storage for the E1-B formulation with a particle size of 5.11 μm compared with 2.98 μm of the control. The other variants (E1-C, E1-D) did not significantly differ on any storage day compared with the control. In E1, the dispersion is highly variable in the measurement stage's intermediate days, especially when FT3-250 is included. This could indicate that the addition of FT3-250 in formulations with 0.72% xanthan gum is not suitable.

**Table 5:** Emulsion preparations (E1, E2, E3) with different proportions of water, xantan gum (XG), sugar and FT3-250.

	**E1**				**E2**				**E3**			
**Ingredient**				%				%				%
Cooking wáter				38.38				38.74				39.1
Egg yolk				4				4				4
Oil				50				50				50
Salt				1.15				1.15				1.15
Xantan gum				0.72				0.36				0
	A	B	C	D	A	B	C	D	A	B	C	D
Sugar	5.75	3.25	2.25	1.25	5.75	3.25	2.25	1.25	5.75	3.25	2.25	1.25
FT3-250	0	2.5	3.5	4.5	0	2.5	3.5	4.5	0	2.5	3.5	4.5

*Emulsions: E (1, 2, 3) indicate variation in percentages of XG (0.72, 0.36, 0%, respectively); (A, B, C, D) indicate the variation in percentages of FT3-250 (A = 0%, B = 2.5%, C = 3.5%, D = 4.5%)*.

Average droplet sizes (d_3,2_) corresponding to the total storage time (21 days) of each emulsion were calculated, and results are presented in Table 6. The average droplet sizes of E1 and E2 (A, B, C, D) are very similar (in ranges of 2.95–3.49 μm). Despite the variation in XG (0.72 and 0.36%, respectively), in E1 and E2, there were no significant differences in averages droplet sizes, independently of the inclusion degree of FT3-250. The emulsion containing no xanthan gum (E3) had the largest droplet sizes, in a range of 4.92–5.19 μm. There were no significant differences in averages droplet size of the E3 (A, B, C, D) independently of the FT3-250 added. Still, significant differences in droplet sizes of E3 compared with E1 and E2 were evidenced (Table 6). Although E3 presented the largest particle sizes, it remained stable over time with minimal particle size variability over time, independently of the inclusion degree for FT3-250 (Figure 1).

**Table 6:** Averages of droplet size (d_3, 2_ μm ) during 21 days of storage of different emulsion formulations.

**Emulsion**	**A**	**B**	**C**	**D**
E1	2.95^*^± 0.23^a^	3.49^*^± 0.78^a^	3.33^*^± 0.52^a^	3.44^*^± 0.25^a^
E2	3.29 ± 0.45^a^	3.32 ± 0.50^a^	3.22 ± 0.43^a^	3.14 ± 0.63^a^
E3	4.92± 0.23^b^	5.11± 0.39^b^	5.19± 0.29^b^	5.19± 0.27^b^

* *Values given are averages of three replicates ± standard deviations. Means within a column or row which are not followed by a common superscript letter (a, b) are significantly different (p < 0.05). E1 (0.72% of XG) E2 (0.36% XG) E3 (0% XG) (A = 0%, B = 2.5%, C = 3.5%, D = 4.5% of FT3-250)*.

#### Creaming Index

The creaming index allows visualiziation of the emulsion's behavior. The higher the creaming index, the greater the instability. Creaming is a problem in O/W emulsions because of the oil droplets being too close to each other (13). Changes in the creaming stability (percentage of the cream layer in the total emulsion) at the end of storage (21 days) of the emulsions (E1, E2, E3) are shown in Table 7. Emulsions (E1 and E2) were highly stable against creaming during 21 days of storage with a creaming index value of 0%; no significant differences were observed compared with respective controls containing only XG (E1-A, E2-A), suggesting that a combination of XG and FT3-250 is an adequate alternative to stabilize the system. On the other hand, the E3 does not contain XG, making it possible to evaluate the emulsion's stability by adding only FT3-250. Results showed that the creaming index of E3 was evidenced in the four formulations (E3-A, B, C, D), but significantly decreased (*p* ≤ *0.05*) in a concentration-dependent manner when FT3-250 was added (Table 7). The control (E3-A) without XG or FT3-250 had a creaming index of 13% while the E3 (B, C, D) with concentrations of 2.5, 3.5, and 4.5% of FT3-250, respectively, had significant lower (*p* ≤ *0.05)* creaming values of 5, 3, 2% respectively.

**Table 7:** Creaming Index of Emulsions (%) at the end of storage period (21 days).

**Emulsion**	**A**	**B**	**C**	**D**
E1	0^*^± 0.0^a^	0^*^± 0.0^a^	0^*^± 0.0^a^	0^*^± 0.0^a^
E2	0 ± 0.0^a^	0 ± 0.0^a^	0 ± 0^a^	0 ± 0.0^a^
E3	14.2 ± 1.7^c^	5 ± 0.8^b^	3 ± 0.7^ab^	2 ± 0.3^a^

* *Values given are averages of three replicates. Means within a column or row which are not followed by a common superscript letter (a, b, c) are significantly different (p < 0.05). E1 (0.72% of XG) E2 (0.36% XG) E3 (0% XG) (A = 0%, B = 2.5%, C = 3.5%, D = 4.5% of FT3-250)*.

#### Rheological Parameters (k and n)

Viscosity is a measure of the fluid's ability to resist deformation. In non-Newtonian fluids are given by the relationship between the shear stress and the strain; within non-Newtonian fluids are pseudoplastic fluids. In assessing the flow index (*n*), a higher value represents a decrease in pseudoplasticity, which means fewer crosslinks are present in the sample (9). On the other hand, viscous nature is represented by the consistency index (*k*); high *k* values indicate high viscosity and stronger and more stable network structure (12). An acceptable salad dressing product with a good mouthfeel should have a low *n* value. On the other hand, a high k value is desirable for the system's stability (9). Our results have shown significant (*p* ≤ 0.05) differences in the *k* values. Higher values of *k* were evidenced for the E1-C and E2-C formulation (XG-0.72-0.36, and 3.5% of FT3-250) compared with their respective controls E1-A and E2-A (Table 8). On the other hand, no significant differences in *k* values were evidenced in the remaining formulations compared with their respective controls. Although there was nosignificant differences for these formulations, the addition of FT3-250 caused an increment of *k* values in a concentration-dependent manner in all cases with a marked tendency toward increasing it. This could be an important finding for the development of new formulations when considering different concentrations of FT3-250 than those mentioned in this study.

**Table 8:** Consistency coefficients (*k*) and flow behavior indices (*n*) for the O/W emulsions with different inclusion degree of FT3-250 and XG.

**Emulsion**	**A**		**B**		**C**		**D**	
	***k* (Pa·sn)^**n**^**	***N***	***k* (Pa·sn)^**n**^**	***n***	***k* (Pa·sn)^**n**^**	***n***	***k* (Pa·sn)^**n**^**	***n***
E1	53.23^*^± 0.02^bc^	0.16^*^± 0.03^a^	100.06^*^± 0.03^ab^	0.17^*^± 0.02^a^	112.51^*^± 0.01^a^	0.19^*^± 0.01^ab^	101.22^*^± 0.02^ab^	0.22^*^± 0.03^ab^
E2	18.24 ± 0.01^d^	0.27 ± 0.04^b^	27.90 ± 0.03^cd^	0.28 ± 0.04^b^	33.63 ± 0.04^c^	0.28 ± 0.02^b^	26.37 ± 0.02^cd^	0.29 ± 0.01^b^
E3	0.82 ± 0.03^e^	0.61 ± 0.01^c^	2.78 ± 0.02^e^	0.53 ± 0.04^c^	4.43 ± 0.03^e^	0.5 ± 0.02^c^	4.77 ± 0.02^e^	0.49 ± 0.04^c^

* *Values given are averages of three replicates. Means within a column or row of the same parameter (k or n) which are not followed by a common superscript letter (a, b, c, d, e) are significantly different (p < 0.05). E1 (0.72% of XG) E2 (0.36% XG) E3 (0% XG) (A = 0%, B = 2.5%, C = 3.5%, D = 4.5% of FT3-250)*.

Otherwise, the values of *n* remained without significant changes in all the formulations compared to their respective controls, but significant differences were evidenced in the *n* values of E3 compared with E1 and E2 (Table 8). The E3 formulation does not contain XG; it only contains FT3-250. This last formulation presented the highest *n* values and the lowest *k* values compared with E1 and E2. This could indicate that the added amounts of FT3-250 are not ideal for the formulation without the addition of XG.

## Discussion

The industry is changing as a result of the continuous improvement of food due to the high demand and the urgency of providing food that contributes to human health. In addition to this, the need to avoid food losses and waste has led to the exploration of new ingredients and processes. These lead us to break paradigms and closely study these new ingredients' behavior and their related products. In this study, we characterize the bromatological content of three different flours obtained from the cob and its rheological behavior to contribute to the utilization of food waste and, at the same time, to provide an added value in the food. The bromatological content of flour varied depending on the cooking treatment. The nutritional content of FT1 was taken as a basis to determine the changes in its content after cooking and to be able to consider the best use of each flour. According to the results presented in Table 1, the ash content of FT2 had a significant decrease of 35.66% compared with the FT3. This could be attributed to the leaching of soluble minerals that could occur in FT2 during cooking, but this does not occur when the cob is cooked with the grain (FT3) due to the presence of the semi-permeable membrane of the grain, which could retain the stored minerals (5). Moncada and Gualdrón de Hernandez (19) reported null migration of minerals after immersion cooking at boiling temperatures on some products with a high degree of hardness, such as the green banana. This condition of hardness in the cob with grain may have contributed to the retention of minerals. It has been reported that corn stores the fat content in the germ (4). When the kernel is separated from the cob, part of the fat content can remain and be quantified in the raw cob, with a value of 1.22% on a dry basis. The flours of the two cooking treatments (FT2 and FT3) showed significantly lower fat values. This could be attributed to the leaching or degradation of this chemical constituent during the cooking process. These results are in accordance with Moncada and Gualdrón de Hernandez (19), who studied the retention of nutrients after cooking high-energy products (potato, yuca, banana) and found that fats are susceptible to deterioration by thermal action. The protein concentrations in FT2 and FT3 had a significant increase compared with FT1 (107.8 and 92.97%, respectively), which may be due to the migration of protein from the germ to the cob since during cooking, this nutrient is adsorbed in the cob structures, which prevents it from being lost, even after drying and grinding (19, 20). Another thing to consider is the loss of water during the drying method with a consequent increase of protein and ash content (20). On the other hand, one of the nutrients most susceptible to immersion cooking is carbohydrates (19). The decrease in total carbohydrates caused by the solubilizaion of some of them in cooking causes an increase in the percentage of other less soluble components, such as protein and crude fiber. This latter represents the insoluble fiber (lignin, cellulose, hemicellulose) and therefore remains on the cob. The differences in crude fiber content in FT2 and FT3 could be the result of leaching of other components, such as soluble fiber, sugars and minerals.

The grinding yield was determined only for the FT3 due to the low content of fat and carbohydrates and high protein content. We obtain a grinding yield of 98.26%, showing very little loss during the grinding process; Dabbour et al. (17), mentions that the grinding time, sieve hole diameter, and moisture are some variables that must be taken into account when processing samples. They reported better grinding yield results in the #40 mesh sieve, with moisture of 10% or less. Our results showed a moisture content of <3% (Table 1), which could have contributed to high grinding performance. In this case, we used a cyclonic mill that had a #40 mesh, and the sample was completely dried. The grinding yield obtained in our process, in which little loss was evidenced, could suggest that mill process factors are well controlled (Table 2). The FT3 was characterized by mechanical granulometry; we obtained four different flours fractions, depending on sieve size (FT3-40, 60, 120, 250), which are classified in Table 3. The greatest retention was obtained in the #40 mesh with a size of 400 μm and diminished as smaller sizes were used. That is to say, the smaller the sieve, the lower the percentage of retention. These results are similar to Córdoba et al. (7), who carried out grinding of ears (#35, 60, 80, and 100 mesh), obtaining four fractions in different percentages (70, 13, 12, and 2%, respectively), obtaining the lowest retention percentage in the smallest size of sieve mesh. The difficulty of obtaining fine particles in the grinding of cob could be due to its structure and abundant fiber composition. Córdoba et al. (7) reported that corn cob's main components are lignin, cellulose, and hemicelluloses, making it difficult to obtain fine particles.

Once the granulometric classification was done, a bromatological analysis of the fractions was carried out. The difference in composition of each fraction was evidenced (Table 4). The content of crude fiber and total carbohydrates showed significant differences among fractions, as did the protein content. According to previous reports, it is possible to use a milling and sieve process to separate flour fractions and concentrate protein, starch, lipids, etc. For example, in the milling of cereals, this type of process helps separate the nutrients required for the different uses. Roa et al. (21) demonstrate that different mill treatments influence the content in amaranth flour fractions with a higher protein concentration, separating it from another that concentrates starch. Masciarelli et al. (22) utilized differential milling to obtain an amaranth flour with a protein content higher than 40% (dry basis). They demonstrated that the moisture content of the grain, temperature, and time of dehydration has an important effect on the flour's protein content. On the other hand, Liu (18) noted that fractions of different particle sizes sieved from the same flour samples varied significantly in chemical composition and reported different protein content of each sieved fraction. These results are in accordance with ours, in which the content of protein, the total carbohydrates and crude fiber varied among fractions. According to our results, the FT3-250 presented the best bromatological characteristics for further analyses since it contains a greater quantity of crude fiber and protein and a lesser quantity of carbohydrates, in addition to having a smaller particle size, desirable for the development of astable emulsion (Table 3). Therefore, dietary fiber was also determined in this fraction, obtaining a greater amount of insoluble fiber. The structural differences are in the types of xylan: being low branched or highly branched, as well as the presence of starches, could be responsible for the differences between crude fiber and soluble and insoluble dietary fiber (23). A disadvantage of the FT3-250 is the low yield obtained (6.81%), which is why the 40 and 60 mesh fractions should be considered for future analysis since they had better performance (62.7 and 22.81%, respectively), representing an alternative as a good source of carbohydrates.

Emulsions were formulated with different amounts of FT3-250 and XG (Table 5). The study of rheological behavior is significant due to the changes that a new ingredient could confer and may affect the shelf life (24). According to Raikos and Ranawana (25), the droplets size is critical for the system's stability and normally is related to the shelf life of O/W emulsions. In this case, the smaller sizes were present in those emulsions that contain a combination of XG and FT3-250. On the other hand, regardless of E3 having the largest particle size, the different concentrations of FT3-250 added did not cause significant variability of the droplet size during storage time, and emulsions (E3-B, C, D) remained significantly stable (*p* ≤ 0.05), compared with control (E3-A) (Figure 1). The emulsions' stability implies prevention of the coalescence, flocculation, and cremation of the droplets since the emulsions are highly dynamic, moving continuously and colliding with each other. These could be influenced by factors such as brownian movement, gravity, or mechanical forces (14, 26). The emulsion stability can be measured through changes in the diameter of the drops over time. This is reported as the coalescence rate and allows the stability of the emulsions to be quantified. Table 9 shows the coalescence rate for the different emulsions (E1, E2, E3). There are no significant differences *(p* ≤ 0.05) in the coalescence rate, despite the degree of inclusion for FT3-250 being evidenced; this is encouraging because it does not cause destabilization; moreover, the lower values of the coalescence rate with exponents −9 and −8 can be classified as very stable (12). Taking this statement into consideration, the E2 (B, C, D) seems to be more stable compared with their control (E2-A) and in addition to the other emulsions, this may suggest that the combination of 0.36% of XG and any inclusion degree of FT3-250 could be optimal to have greater stability.

**Table 9:** Coalescence rate of different emulsion formulation.

**Emulsion**	**A**	**B**	**C**	**D**
E1	^*^2.9667E-7^a^	^*^1.7333E-7^a^	^*^2.6667E-7^a^	^*^3.6667E-7^a^
E2	1.7667E-7^a^	3.000E-8^a^	7.667E-8^a^	3.33E-9^a^
E3	5.000E-8^a^	2.6667E-7^a^	1.9667E-7^a^	1.6667E-7^a^

* *Values given are averages of three replicates. Means within a column or row which are not followed by a common superscript letter (a) is significantly different (p < 0.05). E1 (0.72% of XG) E2 (0.36% XG) E3 (0% XG) (A = 0%, B = 2.5%, C = 3.5%, D = 4.5% of FT3-250)*.

The cremation index is a parameter associated with the system's destabilization and is associated with droplet flocculation (27). Our results showed no creaming index at the end of storage (21 days) for the E1, and E2 formulations, independently of the amount of FT3-250 and/or XG added (Table 7). On the other hand, the E3 formulations (A, B, C, D) without XG presented a creaming process after 21 days of storage. However, significant differences *(p* ≤ 0.05) were evidenced in a concentration-dependent manner when FT3-250 was added, and the creaming index was lower than the control without FT3-250. This diminution in the creaming index could be due to the fiber-rich nature of xylan and starch (7) present in the FT3-250, which help keep the oil droplets separate since the cremation rate is prevented by adding thickening agents such as gums or polysaccharides to reduce the movement of the drops (13, 28, 29). It is demonstrated that O/W emulsions with low pseudoplasticity (*n* ≈ 1) and low viscosity (low *k* value) promote creaminess (9). Our results show that the formulations without XG addition have very low viscosity (low *k* values) and low pseudoplasticity (*n* ≈ 1), and therefore presented creaming. Even though there were no significant changes in the *n* and *k* values when FT3-250 was added, there is a tendency to increase pseudoplasticity and viscosity in a concentration-dependent manner. On the other hand, in the formulations containing the combination of XG and FT3-250, significant differences were evidenced in *n* and *k* values among the formulations E1 -E2 (A, B, C, D), with a tendency toward an increase in viscosity and some cases, significant increase in pseudoplasticity, depending on the amount in the XG-FT3-250 combination. These results are encouraging because the system remains stable despite the addition of FT3-250. These results may suggest a synergistic interaction between XG and FT3-250, contributing to the emulsion's stability. In other research i, this type of synergy has been reported between some ingredients, such as bean gum and XG, among others (9, 30). Such synergy gives us the opportunity, on the one hand, to reduce costs and, on the other, to develop a sustainable product that also provides added value.

## Conclusion

Flours obtained from corn cobs through different cooking treatments (FT2 and FT3) had significant differences in the content of total minerals, crude fiber, and fat. The best cooking treatment to obtain a flour with a higher amount of protein or minerals and less fat or carbohydrates was FT3. The different fractions obtained from FT3 with different sieve mesh sizes varied in their nutrimetal content, the FT3-250 having the highest minerals and crude fiber content. Still, significantly lower fat content and smaller particle size make it suitable for use as a functional ingredient. Emulsions containing XG and FT3-250 seem to cause synergistic interactions, maintain a small particle size, enhance the emulsion stability, and present desirable *n* and *k* values. Despite the nutritional and particle size characteristics of the FT3-250, the yield was low. That is why characterization for the obtained fractions of #40 and #60 mesh should be considered. It is feasible to use the flour as a natural additive to improve added value in dressings. Future studies should be directed toward the characterization of sensory attributes, biological, and other rheological aspects that provide more data on the possible use as a functional ingredient. The data presented here can be used as a basis for the development of new formulations by varying the amount of different ingredients with specific concentrations of xanthan gum.The use of food waste is a current need that will lead us toward a sustainable industry.

## Data Availability Statement

The raw data supporting this article's conclusions will be made available, upon reasonable request, to the corresponding author.

## Author Contributions

KG-A participated in the conception of the idea and provided the resources for the development of the study. KG-A and JB-G participated in the study design and supervised the analyses. AR and NG-S participated in the performance of analyses. SC and ES-G participated in data analysis and collection, as well as writing the original manuscript. MB-V and CG-R performed the statistical analysis. All authors contributed to the article and approved the submitted version.

## Conflict of Interest

The authors declare that the research was conducted in the absence of any commercial or financial relationships that could be construed as a potential conflict of interest.
